# Juvenile dermatomyositis with Anti-SAE antibodies in a Moroccan child associated with pseudo-angioedema: a case report

**DOI:** 10.1186/s12969-023-00921-9

**Published:** 2024-05-21

**Authors:** Khalila Nainia, Mohamed Amine Aouzal, Imane Ouafik, Mariyam Charhbili, Amal Bouchhab, Abdellatif Daoudi, Samira Tizki, Radia Chakiri

**Affiliations:** 1 Pediatrics department, Faculty of Medicine and Pharmacy of Agadir, University Hospital Center SOUSS MASSA Agadir, Ibn ZOHR University, Agadir, Morocco; 2 Pediatrics department, Regional hospital HASSAN II Agadir, Agadir, Morocco; 3 Dermatology department, CHU SOUSS-MASSA, Agadir, Morocco

**Keywords:** Juvenile dermatomyositis, Myositis-specific autoantibody, Pseudo-angioedema, Anti-SAE autoantibody

## Abstract

**Background:**

Juvenile Dermatomyositis (JDM) is the leading cause of non-infectious inflammatory myopathy in children. It is a heterogeneous group of autoimmune diseases characterized by a variable combination of muscular, dermatological, and visceral involvement. Myositis-specific autoantibodies help define homogeneous subgroups with common clinical characteristics and prognoses. Anti-SAE (small ubiquitin-like modifier 1 (SUMO-1) activating enzyme) antibodies are among the most recently discovered specific autoantibodies. The presence of these antibodies is very rare, making it challenging to define clinical features and prognosis in the juvenile form. We report the first case of an African patient with juvenile dermatomyositis and positive anti-SAE antibodies.

**Case Report:**

A 5-year-3-month-old Moroccan boy presented to the pediatric emergency department with dysphagia that had been evolving for two days, preceded two months earlier by facial erythema associated with fatigue, lower limb pain, difficulty walking, and progressive inflammatory polyarthralgia. On admission, the child had a heliotrope rash with predominant pseudo-angioedema on the lips, periungual telangiectasia, and Gottron’s papules over the bilateral interphalangeal and metatarsophalangeal joints. The patient had a more pronounced proximal muscle weakness in the lower limbs. He had no urticaria, fever, arthritis, calcinosis, cutaneous ulcers, or lipodystrophy. The Joint examination was normal, as was the pleuropulmonary examination. The electroneuromyography showed myogenic changes in all four limbs. Laboratory findings showed elevated levels of creatine phosphokinase and lactate dehydrogenase and a mild inflammatory syndrome. The electrocardiogram was normal. The anti-SAE antibodies were positive. The boy was diagnosed with juvenile dermatomyositis. He received methylprednisolone bolus therapy followed by oral prednisone. The latter was gradually tapered in combination with weekly intramuscular methotrexate. As a result, dysphagia disappeared within 48 h. After two weeks, there was an improvement in the muscular score and a significant regression of facial pseudo-angioedema.

**Conclusion:**

We report the first African patient with anti-SAE autoantibody-positive JDM. He had a typical dermatological manifestation of JDM associated with pseudo-angioedema predominant on the lips; a rarely reported sign in DM and JDM patients. The patient responded well to corticosteroid therapy and methotrexate.

## Background

Juvenile Dermatomyositis (JDM) is the leading cause of non-infectious inflammatory myopathy in children. Its annual incidence is estimated to be between 2 and 4 cases per million children [1–5], and its prevalence is 6/100,000 children [6]. The incidence and prevalence rate of JDM in Morocco and Africa is unknown. JDM is a heterogeneous group of autoimmune diseases characterized by a variable combination of muscular, dermatological, and visceral involvement. Myositis-specific autoantibodies help define homogeneous subgroups with common clinical characteristics and prognoses. Anti-SAE (small ubiquitin-like modifier 1 (SUMO-1) activating enzyme) antibodies are among the most recently discovered specific autoantibodies (in 2007) [7]. In adults, this subgroup is characterized by severe dermatological involvement, progressive muscular impairment, dysphagia, fever, and weight loss (8–9). In children, the presence of these antibodies is scarce (< 1%) (10–11), making it challenging to define clinical features and prognosis in the juvenile form. We report the first case of an African patient with juvenile dermatomyositis, positive anti-SAE antibodies, and the only one with pseudo-angioedema.

## Case report

**Fig. 1:**
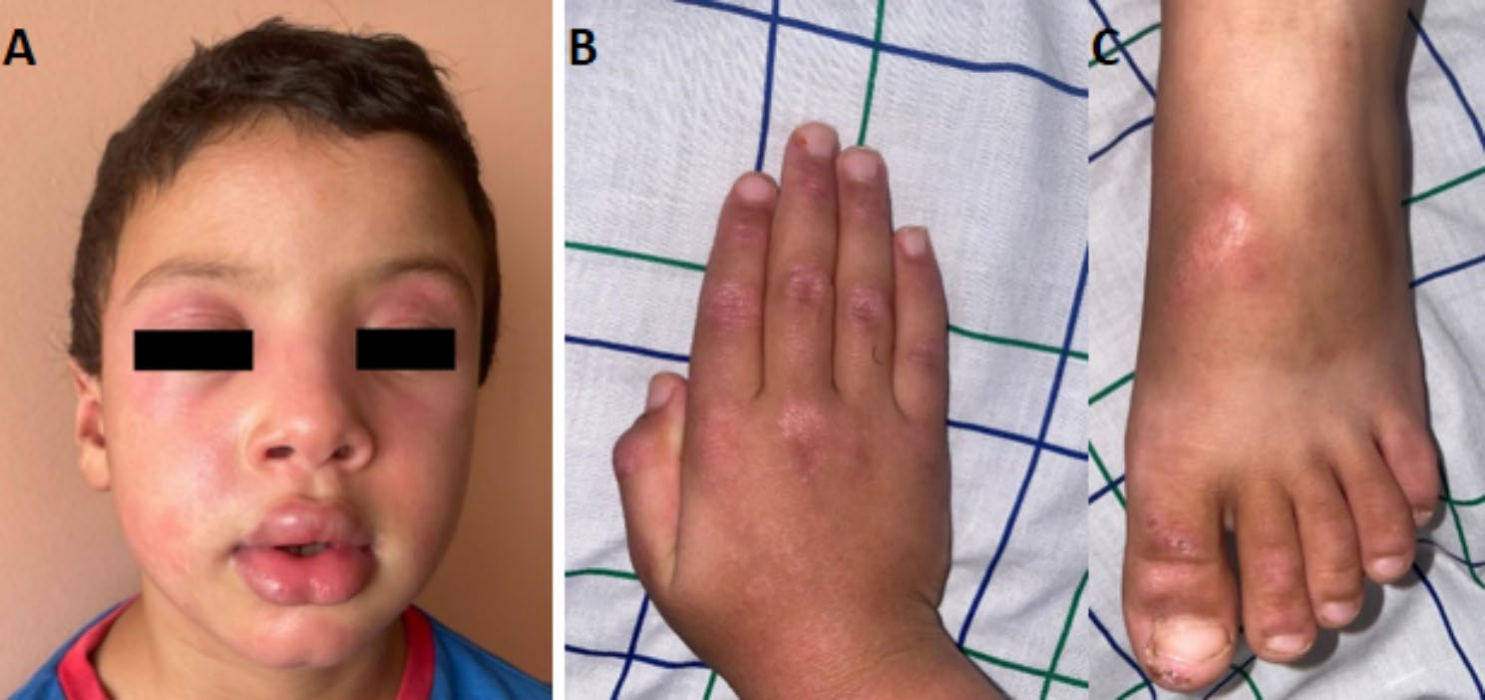
**A** Pseudoangioedema, facial erythema, and heliotrope rash. **B** Erythematous papules over the dorsal side of the interphalangeal and metacarpophalangeal joints of the right hand. **C** Erythematous papules over the dorsal side of the interphalangeal and metatarsophalangeal joints of right feet

Our patient is a 5-year-3-month-old boy. He presented to the pediatric emergency department with dysphagia that had been evolving for two days preceded two months before by fatigue, lower limb pain, difficulty walking, and progressive inflammatory polyarthralgia facial erythema, but without any tingling, itching, or burning sensation. He had no medical or family history of angioedema and had not taken any medication for months before developing the current symptoms. Vital signs were stable on arrival. The child was afebrile. He had a heliotrope rash with facial swelling predominant on the lips, periungual telangiectasia, and Gottron’s papules over the bilateral interphalangeal and metatarsophalangeal joints (Fig. 1). He had more pronounced proximal muscle weakness in the lower limbs (Childhood Myositis Assessment Scale [CMAS] 32/52). He had no urticaria, calcinosis, cutaneous ulcers, or lipodystrophy. Both the joint and the pleuropulmonary examinations were normal.

The electroneuromyography showed myogenic changes in all four limbs. Laboratory findings showed elevated levels of creatine phosphokinase (CPK) and lactate dehydrogenase (LDH) (640 IU/L and 1467 IU/L, respectively) and a mild inflammatory syndrome (erythrocyte sedimentation rate [ESR] = 35 mm and C-reactive protein [CRP] = 24 mg/l). The electrocardiogram was normal. Only anti-SAE antibodies were positive immunoblot. Anti-Mi2 alpha, anti-Mi2 beta, anti-TIF1 gamma, anti-MDA5 and anti-NXP2 antibodies were negative. Serum C1 esterase inhibitor concentration and serum C4 were not dosed.

The diagnosis of juvenile dermatomyositis with anti-SAE was retained according to the criteria of EULAR/ACR 2017 [12].

The patient received methylprednisolone bolus therapy (20 mg/kg/day for three days), followed by oral prednisone at a dose of 2 mg/kg/day. The dose was gradually tapered in combination with weekly intramuscular methotrexate at a dose of 15 mg/m². The oral methotrexate is not available in Morocco. Dysphagia disappeared within 48 h. Two weeks later, there was an improvement in the muscular score (CMAS 47/52) and a significant regression of facial angioedema. However, facial erythema persisted for several months despite local and systemic treatment explained by poor compliance.

## Discussion

Currently, specific myositis antibodies that are identified in dermatomyositis are used to define homogeneous subgroups based on pathophysiological mechanisms, clinical manifestations, paraclinical features, and outcomes guiding treatment selection [10, 13]. Myositis-specific antibodies are found in 30–55% of JDM cases [14–16]. The prevalence of anti-SAE antibodies ranges between < 1% [10] and 7.5% [17] (3/379 in the UK and 4/53 in India). In adults, anti-SAE antibodies are frequently associated with other specific myositis antibodies [18]. However, this association has not been reported in children [17]. This phenotype is characterized by severe and distinctive cutaneous involvement with mild muscular signs in adults [19]. The disease progresses with progressive myopathy, dysphagia, and systemic symptoms (fever, weight loss, and positive inflammation markers) [20–25]. In Asia, it was associated with interstitial lung disease [18, 21, 26]. A UK case series described one child with calcinosis and two with arthritis [10]. However, the rarity of this subgroup in children makes it challenging to define its clinical profile and prognosis. To the best of our knowledge, our patient is the first reported case of anti-SAE-associated JDM in Africa.

Our patient presented with a typical dermatological manifestation of JDM. This was associated with pseudo-angioedema predominant on the lips- a rarely reported sign in DM and JDM patients.

The patient had facial edema, which was most prominent in the lips. The diagnosis of pseudo angioedema associated with juvenile dermatomyositis was carried out for the following reasons: the chronic nature of this edema; the absence of tingling, itching, or burning sensation; the absence of a personal or family history of angioedema; the absence of triggers such as medication; the absence of urticaria; and the presence of associated signs suggestive of JDM [27]. This symptom is not part of the dermatological diagnostic criteria and is rarely reported [28]; however, it may be the first dermatological sign. Ömer Karaca et al. reported two cases of JMD in which angioedema was the only dermatological sign, while the other signs appeared secondarily [29]. They also reviewed the literature for cases of JDM associated with edema. Of the 12 cases associated with edema, seven had generalized edema, one had generalized edema sparing the face, 3 had periorbital edema, and only one had periorbital and upper lip edema. The serologies associated with these phenotypes were not specified [29]. Li Dongmei et al. reported edema in 8 of 76 JDM patients who were anti-NXP2 positive. However, no further information is available concerning the distribution of these edemas [19]. Anti-SAE antibody levels were not specified in most studies reporting this symptom [31–34]. DM presenting with pseudo-angioedema has been reported in roughly 20 cases in adults. One of whom was positive for anti-SAE antibodies [34].

The mechanism of pseudoangioedema in dermatomyositis remains unclear. Several theories are discussed: C1 inhibitor deficiency, Activation of the mast cells, and subcutaneous edema secondary to the extension of inflammation of underlying muscles [29]. The two first mechanisms are common to typical angioedema.

Regarding treatment, Corticosteroids are the first-line treatment for JDM, and methotrexate is recommended for corticosteroid sparing. Other immunosuppressive treatments such as azathioprine, mycophenolate mofetil, cyclosporine, cyclophosphamide, biologics (rituximab), immunoglobulins, and plasmapheresis are considered second and third-line therapies for severe forms, or in case of insufficient response or intolerance to first line treatment [35]. Our patient responded well to corticosteroid therapy. Dysphagia improved within two days, and muscular involvement and facial edema resolved within two weeks. However, facial erythema persisted for several months despite local and systemic treatment explained by poor compliance.

## Conclusion

Juvenile Dermatomyositis with anti-SAE antibodies is a rarely reported entity with a relatively recent description. We report the first case of juvenile dermatomyositis with anti-SAE antibodies in an African patient. He was presented with a typical dermatological manifestation of JDM associated with pseudo-angioedema predominant on the lips. It is a rarely reported sign in DM and JDM patients.

More extensive studies, including multiple populations, are necessary to profile this subgroup better. Such studies will improve our understanding of the role of myositis-specific antibodies, race, age, genetics, and epigenetics in the heterogeneity of dermatomyositis.

## Data Availability

Not applicable.

## Abbreviations

ACR: American college of rheumatology
CMAS: Childhood myositis assessment scale
CPK: Creatine Phosphokinase
CRP: C-reactive protein
DM: Dermatomyositis
ESR: Erythrocyte sedimentation rate
EULAR: European league against rheumatism
JDM: Juvenile dermatomyositis
LDH: Lactate dehydrogenase
MDA5: Melanoma differentiation-associated gene 5
MRI: Magnetic resonance imaging
NXP2: Nuclear matrix protein 2
SAE: Small ubiquitinlike modifier 1 activating enzyme
SUMO-1: Small ubiquitin-like modifier 1
TIF1: Transcriptional intermediary factor 1
UK: United Kingdom
